# Age as a predictive factor for reduced intracranial compliance in patients with headache

**DOI:** 10.1055/s-0044-1779029

**Published:** 2024-02-23

**Authors:** Luiz Gabriel Gonçalves Cherain, Mateus Gonçalves de Sena Barbosa, Ghaspar Gomes de Oliveira Alves Francisco, Luiz Miguel Gonçalves Cherain, Gustavo Frigieri, Nícollas Nunes Rabelo

**Affiliations:** 1 Faculdade Atenas, Passos MG, Brazil.; 2 Centro Universitário de Jaguariúna, Jaguariúna SP, Brazil.; 3 Brain4Care, São Paulo SP, Brazil.

**Keywords:** Intracranial Pressure, Technology, Migraine Disorders, Monitoring, Pressão Intracraniana, Tecnologia, Transtornos de Enxaqueca, Monitoramento

## Abstract

**Background** Increased intracranial pressure (ICP) consists of a set of signs and symptoms related to changes in intracranial compliance (ICC) and ICP.

**Objective** This study presents a retrospective analysis of patients who underwent non-invasive monitoring of ICC based on complaints of headache, correlating decreased brain compliance and increased intracranial pressure.

**Methods** Noninvasive ICC monitoring was performed using a Brain4care device, which contains a strain gauge and a recorder connected to a mechanical device that touches the scalp surface in the frontoparietal area lateral to the sagittal suture. This tool monitors the ICP by identifying small changes in skull measurements that are caused by pressure variations, i.e., skull deformation is associated with the detection of changes in mean ICP. A clinical evaluation of 32 patients with complaints of headache occurred from the analysis of their medical records.

**Results** Of the 32 patients initially chosen, it was possible to complete the analysis of 18 due to the availability of data in the medical records. From the non-invasive monitoring of the ICC, the following data were collected: time-to-peak, P2/P1 ratio, age, and gender. From the statistical analysis of age and P2/P1 ratio, it was noted that as age increases, ICC tends to decrease regardless of sex (p < 0.05).

**Conclusion** This study concluded that there is a correlation between changes in intracranial compliance and headache complaints in outpatients. There was also a relationship between age and decreased intracranial compliance but without a specific pain pattern.

## Resumo

**Antecedentes** O aumento da pressão intracraniana (PIC) consiste em um conjunto de sinais e sintomas relacionados a mudanças na complacência intracraniana (CIC) e na PIC.

**Objetivo** Este estudo apresenta uma análise retrospectiva de pacientes que foram submetidos ao monitoramento não invasivo da CIC com base em queixas de cefaleia, correlacionando a diminuição da complacência cerebral e o aumento da pressão intracraniana.

**Métodos** O monitoramento não invasivo da CIC foi realizado utilizando um dispositivo Brain4Care, que contém um medidor de tensão e um gravador conectado a um dispositivo mecânico que toca a superfície do couro cabeludo na área frontoparietal lateral à sutura sagital. Esta ferramenta monitora a PIC identificando pequenas alterações nas medidas do crânio que são causadas por variações de pressão, ou seja, a deformação do crânio está associada à detecção de alterações na PIC média. Uma avaliação clínica de 32 pacientes com queixas de cefaleia ocorreu a partir da análise de seus prontuários médicos.

**Resultados** Dos 32 pacientes inicialmente escolhidos, foi possível concluir a análise de 18 devido à disponibilidade de dados nos prontuários médicos. A partir do monitoramento não invasivo da CIC, foram coletados os seguintes dados: time-to-peak, relação P2/P1, idade e sexo. Da análise estatística de idade e relação P2/P1, observou-se que à medida em que a idade aumenta, a CIC tende a diminuir independentemente do sexo (p < 0,05).

**Conclusão** Este estudo concluiu que existe uma correlação entre as mudanças na CIC e a queixa de cefaleia em pacientes ambulatoriais. Houve também uma relação entre idade e diminuição da CIC, mas sem um padrão de dor específico.

## INTRODUCTION

Increased intracranial pressure (ICP) is a set of signs and symptoms related to changes in intracranial compliance (ICC) and ICP. These changes are visible in several neurological diseases, including headache. In patients with chronic headache, ICC monitoring is performed in a non-invasive way, which brings less harm to the patient and quicker results. 1

This paper presents a retrospective analysis of patients complaining of headache who were submitted to noninvasive ICC monitoring, and through the monitoring data we can observe patterns of values in P2 and P1 waves, as well as median values of time to peak, which makes it possible to associate headache with increased ICP and decreased ICC. 2

The safest way currently and advocated in this work to measure ICP is through noninvasive devices, as they are safer, have very low rates of post-procedure complications and are also not dependent on the examiner's previous experience. The use of noninvasive devices brings immediacy to the diagnosis of intracranial hypertension and the results of this work demonstrate accuracy in their diagnoses. 2

It is noteworthy that ICP monitoring can be performed by several alternatives, most monitoring is performed with invasive techniques and from the analysis of cerebrospinal fluid (CSF) pressure, these are considered the gold standard and are performed through the insertion of catheters and positioned cranial screws in cranial areas. However, the use of these invasive techniques can lead to some risks of infections and bleeding, which is why noninvasive monitoring is so beneficial to the patient, as there are no risks of these complications, in addition to providing an instant, safe, and comfortable result. for the patient in clinical practice. 3

Finally, the aim of this study is to emphasize that the noninvasive monitoring of ICC allows for quick, safe, and comfortable measurements, together to demonstrate that in patients with headache complaints there is a change in the relationship between the increased value of the P2/P1 wave and to emphasize that these data tend to show an early increase in ICP together with a decrease in ICC.

## METHODS

### Participants

From December 2020 to September 2021, 32 patients seen in the office complaining of headache with nonspecific symptoms and typical migraine without restriction of sex, age, color, race, and socioeconomic status, underwent noninvasive monitoring of intracranial pressure by a Brain4Care device and had their clinical data and results collected. This was an audit of results, and all patients signed a consent form.

### Procedure

Noninvasive monitoring of the patients' intracranial pressure was performed by a Brain4care device, which contains a strain gauge and recorder connected to a mechanical device that touches the scalp surface in the frontoparietal area lateral to the sagittal suture. This tool monitors ICP by identifying small changes in skull measurements that are caused by pressure changes within the skull, i.e., skull deformation is associated with the detection of changes in mean ICP. This procedure is performed with simple, practical handling, it brings efficient results and does not cause complications such as hemorrhages or infections. 2 3 4

### Data collection and outcome measures

The following data were retrospectively collected from patients' medical records: baseline headache characteristics, complaint information, gender, age, previous pathological history, continuous and acute use medications, headache graphs, ICP values, wave morphology, P2/P1 ratio, time-to-peak (TTP) and outpatient consultations.

Each patient underwent a clinical evaluation and ICC monitoring, from which data were generated and collected. In addition, in values above 1.0, the ICP curve suggests changes in intracranial compliance, indicating that such a patient with this result needs constant follow-up, in addition to investigating the underlying cause of this change, which requires individualized treatment and greater attention by the medical team. All medical records were valuable to compare with the results obtained by the sensor.

### Statistics

Descriptive statistics were applied to describe the clinical and demographic characteristics of the patients. Continuous variables were described as mean (standard deviation) or median (interquartile distance), as appropriate under normal data analysis. Categorical data were described as frequencies (valid percentage). To compare means, a T-Student or Mann-Whitney test was applied, as appropriate, for continuous variables. Chi-square test for dichotomous variables. A two-tailed alpha level of 5% was adopted. The analyses were performed using the GraphPad Prism 8.0.0 software (San Diego, USA).

## RESULTS

**Figure 1:**
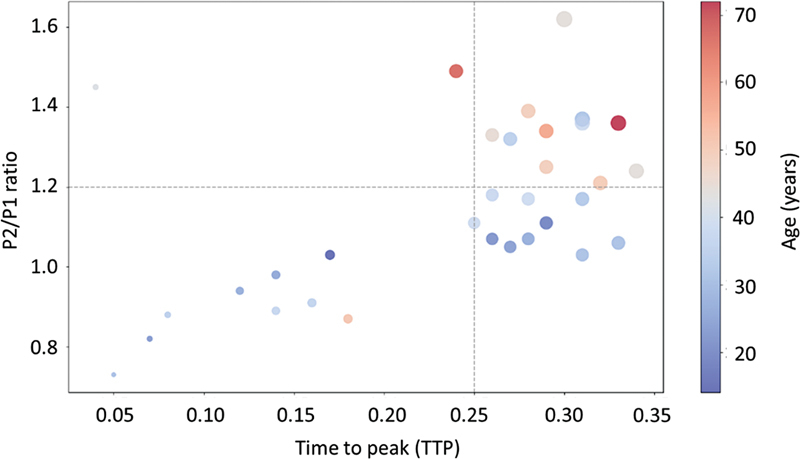
Cluster representing the P2/P1 ratio and the time-to-peak (TTP), to divide four main groups among the four quadrants. Thus, patients in the lower left quadrant tend toward physiological ICC values and in the upper right quadrant toward pathological value.

Of the 32 patients initially chosen, it was possible to complete the analysis of 18 due to the availability of data in medical records. The analyzed patients were divided into a cluster ( Figure 1 ) where the upper right quadrant (URQ group) shows the worst possible clinical condition of intracranial compliance and intracranial pressure, and the lower left quadrant (LLQ group) represents a normal ICP and ICC.

From the noninvasive ICP monitoring, the following data were collected: TTP, P2/P1 ratio, age, and sex. The values found are shown in Table 1 .

**Table 1:** Data from patients in the study, in an assessment of sex, age, time-to-peak and P2/P1 ratio

Group	Patient	Sex	Age	TTP	P2/P1 ratio
LLQ	1	Female	31 y/o	0.05	0.73
LLQ	2	Male	23 y/o	0.07	0.82
LLQ	3	Male	37 y/o	0.14	0.89
LLQ	4	Female	27 y/o	0.12	0.94
LLQ	5	Female	26 y/o	0.14	0.98
LLQ	6	Female	14 y/o	0.17	1.03
LRQ	7	Female	27 y/o	0.28	1.07
LRQ	8	Female	19 y/o	0.29	1.11
LRQ	9	Female	33 y/o	0.31	1.17
LRQ	10	Female	39 y/o	0.28	1.17
URQ	11	Female	50 y/o	0.32	1.21
URQ	12	Male	49 y/o	0.29	1.25
URQ	13	Female	35 y/o	0.27	1.32
URQ	14	Male	45 y/o	0.26	1.33
URQ	15	Male	72 y/o	0.33	1.36
URQ	16	Male	39 y/o	0.31	1.36
URQ	17	Male	49 y/o	0.28	1.39
URQ	18	Female	54 y/o	0.30	1.62

Abbreviations: LLQ, lower left quadrant; LRQ, lower right quadrant; URQ, upper right quadrant; TTP, time-to-peak; y/o, years old.

Patients in the URQ group had the highest P2/P1 ratio, with a mean of 1.35 (range 1.21 - 1.62) and a TTP of 0.29 (range 0.26 - 0.33). The mean age of the patients was also the highest, at 41.1 years old (range 35 - 72). Regarding gender, the URQ group had 5 males and 3 females.

The LLQ group had a P2/P1 ratio of 0.89 (range 0.73 - 1.03) and a TTP of 0.11 (range 0.05 - 0.17). The mean age of the patients was 26.3 years old (range 14 - 37). It was observed that in this group there were 4 females and 2 males. This group had normal TTP and P2/P1 ratio values, despite migraine complaints.

From the analysis of the LRQ group, in which patients reported migraine symptoms but with ICP and ICC in their normal parameters, a mean P2/P1 ratio of 1.13 (range 1.07 - 1.17) and TTP of 0.29 (range 0.28 - 0.31) were found. The mean age was 29.5 years old (range 19 - 39).

**Figure 2:**
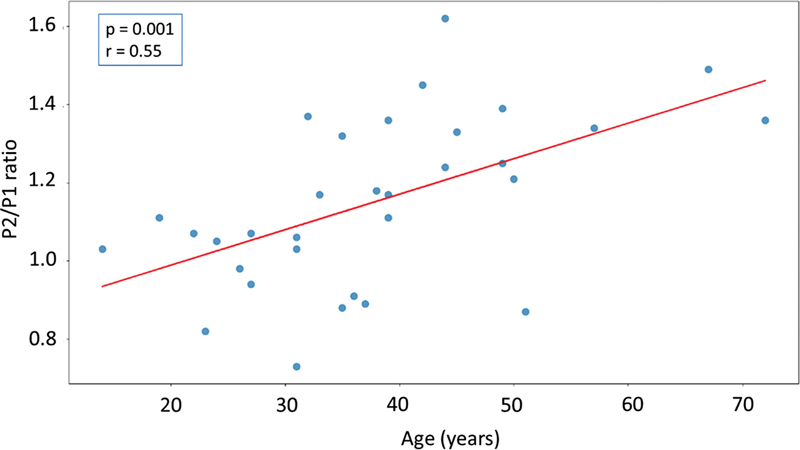
Statistical analysis of age and P2/P1 ratio.

From the statistical analysis of age and the P2/P1 ratio ( Figure 2 ), it was possible to notice that as age increases, intracranial compliance tends to decrease independent of gender (p < 0.05).

When evaluating the patients' symptomatology, diseases, and comorbidities, in the URQ group there was one case of Arnold's neuralgia and the others, symptoms characteristic of migraine and severe headache. The LLQ group also had two cases of Arnold's neuralgia, one case of anxiety crisis associated with migraine, and one case of associated essential tremor. In the LRQ group, there was one presentation of anxiety, hypertension, hypothyroidism, and intrauterine contraceptive use related to the headache complaint. There was also one report of chronic headache after traumatic brain injury and one case of past seizure.

**Figure 3:**
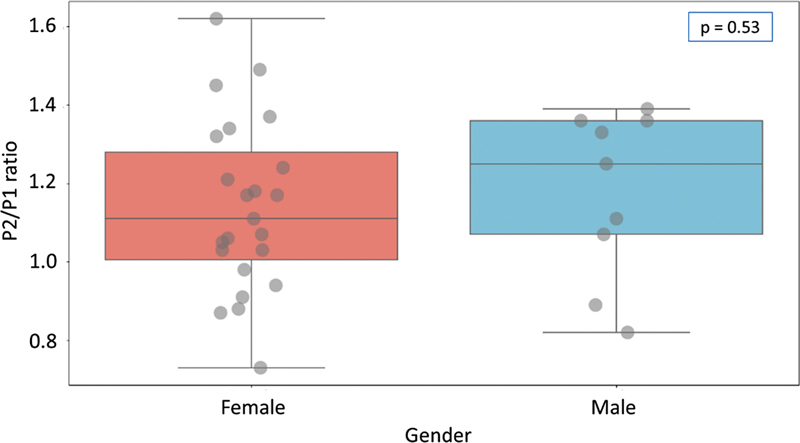
Statistical analysis of gender and P2/P1 ratio.

Despite all reported diseases and comorbidities, all groups had characteristic migraine symptoms and similar clinical conditions. It was possible to observe a male dominance in migraine complaints in the URQ group, to note that possibly male patients tend to have headache associated with decreased ICC and elevated ICP, while females have headache complaints with both altered values and values within the normal range. Although clinically important, was statistically insignificant (p > 0.05) in the analysis, as shown in Figure 3 .

To infer the treatment, the association between the drugs propranolol and topiramate was used most often, with effective results in the symptomatology of patients and subsequent weaning from topiramate. Neurolytic blockade was requested in cases of Arnold's neuralgia in addition to drug treatment. The use of duloxetine, lamotrigine, and pregabalin was also prescribed in isolated cases where there was no improvement with the usual treatment.

## DISCUSSION

The main finding of the study suggests that high and low ICP may lead the patient to the complaint of headache, possibly due to changes in ICC. Age was directly related to decreased ICC in the study patients. An interesting finding was high ICP related to headache complaints by male patients, while female patients had complaints at a variety of pressures assessed, including within normal values. Male patients represent 40% of our data but seem to have stronger and more challenging headache ( Table 2 ).

**Table 2:** Characteristics of patients categorized by sex and P2/P1 ratio

Sex	Patients N (%)	P2/P1 ratio < 1.2 N (%)	P2/P1 ratio > 1.2 N (%)
**Female**	11 (61.1)	8 (80)	3 (37.5)
**Male**	7 (38.8)	2 (20)	5 (62.5)
**Total**	18 (100)	10 (100)	8 (100)

The noninvasive system was important in observing homeostasis in the intracranial compartments, especially through the physician's office, where analysis tools are usually limited. The values obtained from the ICP curves are accurate in predicting intracranial hypertension (P2/P1 ratio > 1.2) in patients with cranial integrity. 5

Headaches without specific clinical signs or imaging features can be challenging for neurologists and neurosurgeons regarding diagnosis, especially in idiopathic intracranial hypertension or intracranial hypotension. The chronicity of the disorders leads to a tendency for the typical symptoms to diminish, remaining only headache as the main complaint. The elderly require special attention because of their physiologically decreased ICC. In this sense, noninvasive monitoring in the physician's office is important for the differential diagnosis of the disease, especially when conventional treatments do not work. 6 7 8

Studies have attempted to search for a relationship between comorbidities and the occurrence of headache. It has been found that there is a comorbid relationship between idiopathic intracranial hypertension without papilledema and chronic migraine, through trigeminovascular innervation of the Dural sinuses and cortical bridge veins, which acts as an “intracranial pressure sensor”, and may represent an important source of calcitonin gene-related peptide (CGRP) and cause severe headaches. 9

Most patients complain of a progressive worsening of headache over weeks, but some may develop a rapid increase in ICP in a short period of time. There may be reports of headache with pulsatile features, which may be aggravated by physical activity, coughing, and exertion. It can also be noted that headache due to elevated ICP mimics some diseases, such as chronic migraine and tension headache. Undiagnosed elevated ICP can lead to irreversible consequences, such as neurological deficits, blindness, and in the most severe cases, death. 10 11 12

The noninvasive method of monitoring ICP and ICC stands out over traditional invasive methods because it is relatively cheaper, does not require an experienced neurosurgical team (monitoring can be done anywhere and requires only basic training in the use of the equipment), and lastly, does not present risks to the patient, such as hemorrhage and infection. Importantly, noninvasive monitoring can also be used in patients at risk of ICC disturbance who cannot undergo ICP catheter placement, such as sepsis, liver failure, and severe acute respiratory syndrome. 5 13 14

The study has some limitations, such as the absence of a control group. Also, the focus of the analysis started from the headache symptom, without considering the diagnosis.

Therefore, the study concludes that there is a correlation between changes in intracranial compliance and complaints of headache in outpatients. There was also a relationship between age and decreased intracranial compliance in the context of headache, but without a specific pain pattern. Further studies should be conducted to confirm this association. The use of noninvasive ICP and ICC monitoring is effective in predicting intracranial hypertension and changes in cerebral hydrodynamics.
